# Associations of polymorphisms in the myeloid immunoregulatory receptor gene MRC1 with expression and prognosis in acute myeloid leukemia

**DOI:** 10.3389/fimmu.2026.1949691

**Published:** 2026-09-07

**Authors:** Meng Li, Fangjing Yin, Zhiyu Shi, Tao Sun, Chunyan Ji, Mingying Li

**Affiliations:** 1 Shandong Key Laboratory of Hematological Diseases and Immune Microenvironment, Department of Hematology, Qilu Hospital of Shandong University, Jinan, Shandong, China; 2 Department of Hematology, Qilu Hospital of Shandong University, Cheeloo College of Medicine, Shandong University, Jinan, Shandong, China

**Keywords:** acute myeloid leukemia, gene expression, MRC1, overall survival, single-nucleotide polymorphism

## Abstract

**Background:**

Acute myeloid leukemia (AML) shows substantial biological and clinical heterogeneity that extends beyond leukemia-intrinsic genetic alterations. MRC1, which encodes the myeloid receptor CD206, has been linked to monocytic differentiation and immunoregulatory states in AML. We investigated whether the MRC1 variants rs691005 and rs2253120 are associated with AML occurrence, development and prognosis.

**Methods:**

We genotyped rs691005 and rs2253120 in 335 patients with AML and 326 healthy controls. Their associations with AML susceptibility, cytogenetic abnormalities, risk stratification, complete remission after two treatment cycles, and overall survival (OS) were examined under co-dominant, dominant, and recessive models. Independent prognostic effects were assessed using multivariable Cox regression. The relationship between SNP and MRC1 expression was evaluated using GTEx eQTL data and genotype-stratified expression measurements in primary AML bone marrow CD34^+^ cells. TCGA datasets were used to assess MRC1 expression and survival in AML. Virtual MRC1 knockdown and pathway analysis was performed using a single-cell RNA-sequencing dataset generated at our center.

**Results:**

Neither variant was associated with AML susceptibility, cytogenetic abnormalities, or early remission. rs691005 was associated with adverse risk stratification and showed a nominal association with shorter OS in Kaplan-Meier analysis. rs2253120 AA homozygosity was associated with improved OS after multivariable adjustment and was linked to lower MRC1 expression in GTEx and primary AML samples. Higher MRC1 expression was associated with poorer OS in TCGA-LAML. Virtual MRC1 knockdown revealed exploratory enrichment patterns related to myeloid differentiation, adhesion, cytokine production, and immune regulation.

**Conclusions:**

MRC1 rs691005 was linked to adverse risk stratification and shorter OS, whereas rs2253120 AA homozygosity was independently associated with better OS and lower MRC1 expression. These findings support a potential role for inherited variation at the MRC1 locus in the clinical and immune heterogeneity of AML.

## Introduction

Acute myeloid leukemia (AML) is shaped by recurrent genetic and epigenetic alterations that define biologically and clinically distinct disease subgroups (1). However. these leukemia-intrinsic abnormalities do not fully account for the marked heterogeneity of the disease. Patients with similar genomic backgrounds may differ substantially in chromatin state, differentiation trajectory, and malignant cellular composition, each of which can carry distinct clinical implications (1, 2). Among these cellular states, differentiated monocyte-like AML cells are particularly notable because they express broad immunoregulatory programs and can directly suppress T-cell activity *in vitro* (2). More recent single-cell and multimodal studies have further linked immune-cell differentiation and dysfunction to treatment resistance and adverse survival in AML (3). These observations indicate that immune escape in AML is likely shaped by multiple layers of regulation, including genetic variation, cellular differentiation, and immune-cell interactions.

Mannose receptor C-type 1 (MRC1), also known as CD206, encodes a transmembrane C-type lectin receptor that recognizes glycosylated ligands and mediates their internalization and intracellular processing (4). Although CD206 is widely used as a marker of alternatively activated or M2-like macrophages, it does not define a single, fixed macrophage phenotype. In addition to ligand uptake, MRC1 is also involved in antigen processing, inflammatory signal transduction and the regulation of immune cell responses (5, 6). Furthermore, its role in cancer also depends on the environment. Studies have shown that CD206-positive myeloid cells are usually associated with an immunosuppressive tumor microenvironment, but under certain conditions, they may also support antigen presentation and anti-tumor T-cell responses (7). In conclusion, these findings suggest that the expression of MRC1 should be interpreted in the context of cell type and tumor environment, rather than being regarded as a fixed marker of immunosuppression.

This distinction is particularly significant in AML, where MRC1 is expressed both by bone marrow macrophages and by malignant cell subpopulations characterized by monocytes (2, 8). Single-cell transcriptomic studies have placed MRC1 within an immunomodulatory gene program expressed by monocyte-like AML cells capable of suppressing T-cell activation (2). An MRC1/CD36-positive, M2-like macrophage state has also been identified in AML bone marrow and linked to increased PD-L1 expression and impaired CD8^+^ T-cell function (9). Increased numbers of CD206-positive myeloid cells and higher MRC1 expression have likewise been associated with adverse clinical outcomes in AML (10). In addition, MRC1 can be detected on leukemic blasts in a subset of patients while remaining low in normal hematopoietic progenitor cells (11). Collectively, these observations position MRC1 at the intersection of monocytic differentiation, immune regulation, and clinical behavior in AML. However, the factors that lead to significant differences among patients remain poorly understood.

Inherited genetic variation may account for part of this variability. Single-nucleotide polymorphisms (SNPs) can influence gene expression by altering transcription, RNA processing, transcript stability, or translation, or by tagging nearby functional variants through linkage disequilibrium (12). These regulatory effects are often highly dependent on tissue, cell type, and cellular state (13). The MRC1 variants rs2253120 and rs691005 have previously been examined in sarcoidosis, and rs691005 has also been associated with treatment response in hepatitis C virus infection, suggesting that inherited variation at this locus may be relevant to immune-related phenotypes (14, 15). Public eQTL data further link rs2253120 to MRC1 expression in whole blood and other tissues (13). Whether these two variations are related to MRC1 expression or AML outcomes, as well as which cellular programs are associated with MRC1 activity, remains unresolved.

Based on our earlier work, we focused on rs2253120 and rs691005, and investigated their relationships with AML susceptibility, risk stratification, complete remission after two treatment cycles, and overall survival rate under multiple genetic models. Then, we combined the publicly available eQTL data with genotype-stratified MRC1 expression measurements in primary AML bone marrow samples and evaluated the prognostic significance of MRC1 expression in an independent transcriptomics cohort. Finally, using the single-cell RNA sequencing dataset generated by our center, we conducted virtual MRC1 knockdown and then carried out pathway analysis to explore the cellular programs associated with the decreased expression of MRC1. This comprehensive approach enables us to examine whether the genetic variations at the MRC1 locus are associated with gene expression and clinical outcomes in the context of disease-related bone marrow immunity.

## Methods

### Study subjects

**Figure 1:**
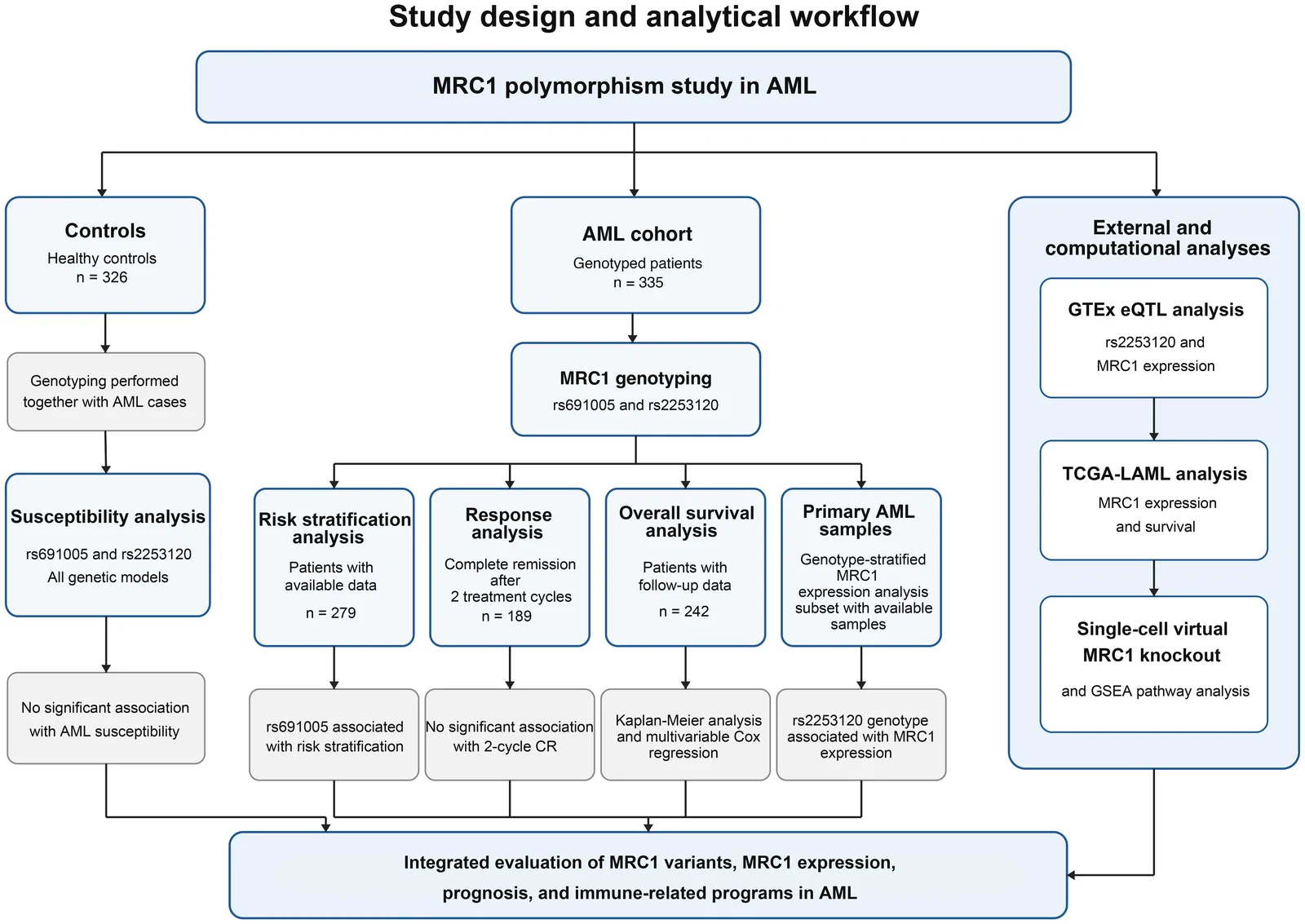
Study design and analytical workflow. A total of 335 patients with AML and 326 healthy controls were genotyped for MRC1 rs691005 and rs2253120. The analyses included AML susceptibility, risk stratification (*n* = 279), complete remission after two treatment cycles (*n* = 189), overall survival (*n* = 242), and genotype-stratified MRC1 expression in primary AML samples. Public datasets were used to assess the rs2253120-MRC1 eQTL relationship and the expression and prognostic relevance of MRC1. Virtual MRC1 knockdown and gene set enrichment analysis were performed using a single-cell RNA-sequencing dataset generated by our group. CR, complete remission; eQTL, expression quantitative trait locus; GSEA, gene set enrichment analysis; TCGA-LAML, The Cancer Genome Atlas acute myeloid leukemia cohort.

A total of 335 patients with AML and 326 healthy controls were enrolled in the study. AML was diagnosed in accordance with the National Comprehensive Cancer Network (NCCN) guidelines. Baseline demographic and clinical characteristics of the study participants are presented in **Table 1**, and an overview of the study design, patient cohorts, and analytical workflow is provided in **Figure 1**. Due to the retrospective nature of the study, different analyses were performed in partially overlapping subsets according to data availability. Patients with missing relevant clinical information were excluded from the corresponding analyses. As acute promyelocytic leukemia (AML-M3) differs substantially from other AML subtypes in terms of molecular characteristics, treatment strategies, and clinical outcomes, subsequent analyses focusing on risk stratification, treatment response, and survival were restricted to non-M3 AML patients. Chemotherapy status was defined according to whether patients received standard anti-leukemic treatment after diagnosis. The study was conducted in accordance with the Declaration of Helsinki and was approved by the Medical Ethics Committee of Qilu Hospital of Shandong University. Written informed consent was obtained from all participants or their legally authorized representatives before enrollment.

**Table 1:** Demographic and clinical characteristics.

Characteristic	Case n (%)	Control n (%)
Age (years, median, range)	48 (13–90)	40 (20–90)
< 60	257 (77)	302 (93)
≥ 60	78 (23)	24 (7)
Gender		
Male	180 (54)	119 (37)
Female	155 (46)	207 (63)
FAB classification		
M3	56 (17)	n.a.
Non-M3	279 (83)	n.a.
WBC^*^		
< 100	225 (81)	n.a.
≥ 100	54 (19)	n.a.
Risk stratification^*^		
Favorable	59 (21)	n.a.
Intermediate	127 (46)	n.a.
Adverse	93 (33)	n.a.
Response^#^		
CR	145 (77)	n.a.
No CR	44 (23)	n.a.

^*^Non-M3 patients (n=279).

^#^Non-M3 patients evaluated after 2 cycles of treatment (n=189)

n.a., not applicable.

### DNA extraction and genotyping

Genomic DNA was isolated from bone marrow mononuclear cells (BMMCs) using the TIANamp Blood DNA Kit (Tiangen Biotechnology, China) according to the manufacturer’s instructions. SNP genotyping was performed using the Sequenom iPLEX and MALDI-TOF-based MassARRAY platform (BGI Tech, Beijing, China). The platform is based on multiplex PCR, site-specific single base extension reactions, and MALDI-TOF spectroscopy.

### RNA extraction and real-time PCR

MRC1 mRNA expression was evaluated in matched bone marrow specimens obtained from patients included in the SNP genotyping cohort. Due to limited availability of qualified archived bone marrow samples, 17 patients with available matched specimens were included in the expression analysis, consisting of 6 GG, 7 GA, and 4 AA genotype carriers of rs2253120. Bone marrow mononuclear cells (BMMCs) were isolated from the matched bone marrow specimens, and CD34^+^ cells were subsequently enriched using a EasySep Human CD34 Positive Selection Kits (STEMCELL Technologies) according to the manufacturer’s instructions. Total RNA was isolated from the isolated CD34^+^ cells using the TRIzol reagent (Invitrogen), and cDNA was synthesized using PrimeScript™ RT Master Mix (Takara). Quantitative PCR was performed using the TB Green^®^Premix Ex Taq™ II (Takara). The 2^-ΔCT^ method was used to analyze the results. The expression data were normalized to the expression of GAPDH.

### Public dataset analysis

Publicly available transcriptomic data were analyzed using the Gene Expression Profiling Interactive Analysis (GEPIA, http://gepia.cancer-pku.cn) platform, which integrates RNA sequencing data from The Cancer Genome Atlas (TCGA) and the Genotype-Tissue Expression (GTEx) projects. MRC1 expression levels were compared between AML samples from the TCGA-LAML cohort and normal control samples from the GTEx dataset. The normal control group consisted of 70 whole blood samples. The association between MRC1 expression and overall survival was evaluated using Kaplan-Meier survival analysis based on TCGA-LAML clinical data.

### In silico MRC1 knockdown

The virtual knockdown analysis was designed as an exploratory computational perturbation approach to investigate potential cellular programs associated with reduced MRC1 expression. The normalized single-cell expression matrix was used to simulate MRC1 knockdown by reducing MRC1 expression to 20% of its baseline level, corresponding to an 80% simulated reduction. This degree of perturbation was selected to represent a substantial reduction while maintaining residual expression variability for downstream analysis. Spearman correlation coefficients were calculated between MRC1 expression and all expressed genes across cells. The 30 genes showing the strongest positive correlations and the 30 genes showing the strongest negative correlations were retained to define the MRC1-associated co-expression network, balancing the inclusion of strongly associated genes with reduction of potential noise. Perturbation effects were propagated according to both the direction and magnitude of gene correlations. Positively correlated genes were downregulated, whereas negatively correlated genes were upregulated. The magnitude of each propagated effect was weighted by the normalized absolute Spearman correlation coefficient and moderated using a scaling factor of α=0.35 to control the extent of perturbation. These perturbation values were added to the original expression matrix to generate a simulated post-knockdown expression matrix. Gene set enrichment analysis (GSEA) and related pathway enrichment analyses were performed using R software.

### Statistical analysis

The Hardy-Weinberg equilibrium (HWE) of genotype distributions in the control group was assessed using the chi-square test. The associations between MRC1 SNPs and AML susceptibility, risk stratification, and response to induction chemotherapy were evaluated using the chi-square test. Binary logistic regression and ordinal logistic regression analyses were performed to estimate age- and sex-adjusted odds ratios (ORs) and corresponding 95% confidence intervals (CIs). To account for multiple comparisons, the Benjamini-Hochberg false discovery rate (FDR) method was applied to SNP association analyses involving categorical clinical endpoints. FDR adjustment was performed separately for each endpoint, including six comparisons per endpoint based on two SNPs and three genetic models (co-dominant, dominant, and recessive). For *p* values reported by SPSS as *p* < 0.001, a value of 0.001 was conservatively used for FDR calculation. Overall survival analyses were considered prespecified prognostic analyses and were evaluated using Kaplan-Meier and multivariable Cox regression models with adjustment for established prognostic factors. All statistical analyses were conducted using SPSS 27.0 software (SPSS Inc., Chicago, IL, USA). A two-sided *p* value <0.05 was considered statistically significant.

## Results

### Study population and SNP quality control

The study included 335 patients with AML and 326 healthy controls (**Table 1**). The median age was 48 years (range, 13–90 years) in the AML group and 40 years (range, 20–90 years) in the control group. Among AML patients, 180 (54%) were male and 155 (46%) were female, whereas the control group comprised 119 (37%) males and 207 (63%) females. Significant differences in age and sex distribution were observed between the two groups, and these variables were adjusted as covariates in subsequent analyses. Among AML patients, 56 (17%) were diagnosed with acute promyelocytic leukemia (M3), while 279 (83%) had non-M3 AML. Within the non-M3 AML subgroup, 59 (21%) patients were categorized as favorable risk, 127 (46%) as intermediate risk, and 93 (33%) as adverse risk according to risk stratification criteria. Among the 189 patients assessed after two cycles of induction chemotherapy, 145 (77%) achieved complete remission (CR), whereas 44 (23%) failed to achieve CR.

Both MRC1 SNPs (rs691005 and rs2253120) satisfied the predefined quality control criteria (**Table 2**). The MAFs among healthy controls were 34.36% and 20.86%, respectively. No deviation from Hardy-Weinberg equilibrium was observed for either SNP in the control group.

**Table 2:** Selected genes and SNPs.

Gene	SNP	Variant	Variant allele	MAF	HWE (*p*-value)
MRC1	rs691005	T>C	C	34.355828221%	0.932575529
	rs2253120	G>A	A	20.858895706%	0.963222487

SNP, single nucleotide polymorphisms.

### Relationship between MRC1 gene polymorphism and AML susceptibility

We used three gene models (co-dominant, dominant and recessive) to analyze the relationship between MRC1 SNPs and AML susceptibility. Genotype distributions were initially compared using the chi-square test or Fisher’s exact test. No significant associations were identified between either MRC1 SNP and AML susceptibility under any genetic model (*p*>0.05; **Table 3**), suggesting that MRC1 SNPs were not associated with AML susceptibility.

**Table 3:** Association between MRC1 gene polymorphism and AML susceptibility.

Gene	SNP	Model	Genotype	Control (n)	AML (n)	*x*2 test *p* value	OR (95% CI)	Adjusted *p* value
MRC1	rs691005	Co-dominant	TT	142	145	0.788		
			CT	144	143		0.998 (0.710-1.404)	0.992
			CC	40	47		1.002 (0.604-1.662)	0.994
		Dominant	TT	142	145	0.943		
			CT+CC	184	190		0.999 (0.725-1.377)	0.995
		Recessive	TT+CT	286	288	0.503		
			CC	40	47		1.003 (0.623-1.615)	0.991
	rs2253120	Co-dominant	GG	205	194	0.411		
			GA	106	125		1.288 (0.914-1.795)	0.151
			AA	15	16		1.068 (0.494-2.312)	0.867
		Dominant	GG	205	194	0.191		
			GA+AA	121	141		1.254 (0.906-1.736)	0.172
		Recessive	GG+GA	311	319	0.915		
			AA	15	16		0.976 (0.456-2.091)	0.950

SNP, single nucleotide polymorphisms; AML, acute myeloid leukemia; OR, odds ratio; CI, confidence interval.

### Association between MRC1 gene polymorphism and cytogenetic abnormalities

Among the 335 AML patients, 279 were diagnosed with non-M3 AML. Because M3 subtype differs from other AML subtypes in terms of treatment strategies and clinical outcomes, only patients with non-M3 AML were included in the subsequent analyses. We then evaluated the relationship between MRC1 gene polymorphisms and cytogenetic abnormalities. Neither rs691005 nor rs2253120 was associated with AML karyotype status (*p* > 0.05; data not shown), indicating that these variants were not linked to conventional cytogenetic abnormalities.

### Association between MRC1 gene polymorphism and baseline peripheral-blood characteristics

We further investigated whether MRC1 gene polymorphisms were associated with baseline peripheral-blood characteristics, including WBC count, HGB level, and PLT count. In this study, the high WBC group was defined as patients with WBC count ≥100×10^9^/L, whereas the low WBC group included patients with WBC count <100×10^9^/L. The high PLT group was defined as PLT levels >50×10^9^/L, and the low PLT group as PLT levels ≤50×10^9^/L. The high HGB group included patients with HGB levels ≥60 g/L, while the low HGB group included patients with HGB levels <60 g/L. However, neither SNP showed a significant association with these peripheral-blood parameters **(Supplementary Tables 1**–**3**).

### MRC1 rs691005 is associated with AML risk stratification

According to the NCCN Clinical Practice Guidelines, AML patients were classified into favorable-, intermediate-, and adverse-risk groups based on molecular and cytogenetic abnormalities. We further investigated the association between MRC1 SNPs and AML risk stratification. We conducted a preliminary screening by chi-square test or Fisher exact test under the above three models. As shown in **Table 4**, MRC1 rs691005 under co-dominant and dominant models were significantly associated with the AML risk stratification (*p* = 0.002 and *p* < 0.001, respectively). Subsequently, multivariate ordinal logistic regression analyses adjusted for age and sex were conducted to further evaluate this association. The TC and CC genotype of MRC1 rs691005 under co-dominant model (OR = 2.472, 95% CI = 1.501-4.067, *p* < 0.001; OR = 2.751, 95%CI=1.401-5.409, *p* = 0.003), and the TC/CC genotype of MRC1 rs691005 under dominant model (OR = 2.550, 95% CI = 1.606-4.046, *p* < 0.001) tended to be associated with increased odds of higher-risk AML stratification. To account for multiple comparisons, we further performed FDR correction. The association between MRC1 rs691005 and AML risk stratification remained significant after FDR adjustment under both the co-dominant model and the dominant model (FDR-adjusted *p* = 0.006, both; **Supplementary Table 5**). In contrast, no significant association was observed between MRC1 rs2253120 and AML risk stratification.

**Table 4:** Association between MRC1 gene polymorphism and AML risk stratification.

Gene	SNP	Model	Genotype	Favorable	Intermediate	Adverse	*x*2 test *p* value	OR (95% CI)	Adjusted *p* value
MRC1	rs691005	Co-dominant	TT	32	65	25	**0.002**		
			TC	22	44	48		2.472 (1.501-4.067)	**< 0.001**
			CC	5	18	20		2.751 (1.401-5.409)	**0.003**
		Dominant	TT	32	65	25	**< 0.001**		
			TC+CC	27	62	68		2.550 (1.606-4.047)	**< 0.001**
		Recessive	TT+TC	54	109	73	0.083		
			CC	5	18	20		1.817 (0.969-3.401)	0.063
	rs2253120	Co-dominant	GG	36	69	60	0.543		
			GA	21	50	30		0.915 (0.573-1.461)	0.710
			AA	2	8	3		0.622 (0.213-1.815)	0.385
		Dominant	GG	36	69	60	0.299		
			GA+AA	23	58	33		0.873 (0.557-1.369)	0.554
		Recessive	GG+GA	57	119	90	0.493		
			AA	2	8	3		0.642 (0.223-1.850)	0.412

SNP, single nucleotide polymorphisms; OR, odds ratio; CI, confidence interval.

The bold values mean statistically significant p-values.

### The relationship between MRC1 SNPs and response of induction chemotherapy

Among the enrolled 279 non-M3 AML patients, 230 patients received induction chemotherapy. After two cycles of induction therapy, BM cytological evaluation was performed in 189 patients, of whom 145 (77%) achieved complete remission (CR), while 44 (23%) failed to achieve CR. We analyzed the associations between MRC1 SNPs and induction chemotherapy response using chi-square or Fisher’s exact tests under different genetic models (**Table 5**). However, there were no significant differences between MRC1 variants and response of induction chemotherapy.

**Table 5:** Association between MRC1 SNPs and response of induction chemotherapy.

Gene	SNP	Model	Genotype	No CR	CR	*x*2 test *p* value	OR (95% CI)	Adjusted *p* value
MRC1	rs691005	Co-dominant	TT	19	70	0.394		
			CT	21	54		0.582 (0.275-1.231)	0.156
			CC	4	21		1.712 (0.504-5.823)	0.389
		Dominant	TT	19	70	0.553		
			CT+CC	25	75		0.755 (0.376-1.516)	0.430
		Recessive	TT+CT	40	124	0.355		
			CC	4	21		2.149 (0.667-6.931)	0.200
	rs2253120	Co-dominant	GG	26	87	0.944		
			GA	16	53		0.938 (0.454-1.936)	0.862
			AA	2	5		1.146 (0.195-6.742)	0.881
		Dominant	GG	26	87	0.914		
			GA+AA	18	58		0.958 (0.476-1.929)	0.905
		Recessive	GG+GA	42	140	0.665		
			AA	2	5		1.171 (0.203-6.767)	0.860

SNP, single nucleotide polymorphisms; No CR, non-remission; CR, complete remission; OR, odds ratio;.

CI, confidence interval.

### MRC1 rs2253120 and rs691005 were associated with AML overall survival

**Figure 2:**
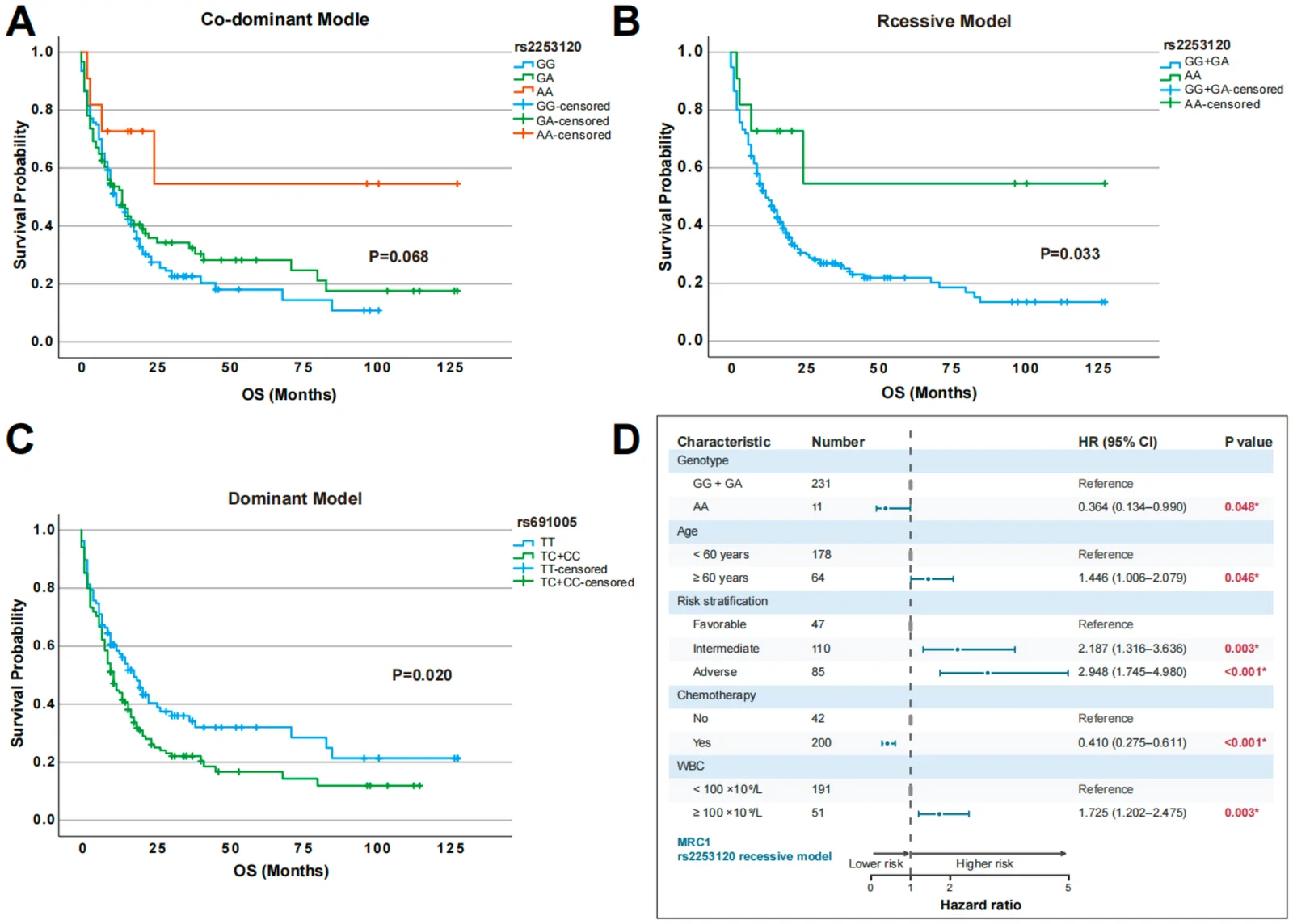
Associations of MRC1 polymorphisms with OS in AML patients. **(A)** Kaplan-Meier OS curves stratified by the MRC1 rs2253120 genotypes GG, GA, and AA under the co-dominant model. **(B)** Kaplan-Meier OS curves for rs2253120 under the recessive model, comparing patients with the AA genotype with those carrying the GG/GA genotypes. **(C)** Kaplan-Meier OS curves for rs691005 under the dominant model, comparing patients with the TT genotype with those carrying the TC/CC genotypes. **(D)** Forest plot of the multivariable Cox proportional hazards analysis under the rs2253120 recessive model.

We used three genetic models to analyze the association between MRC1 SNPs and overall survival (OS) in 242 non-M3 AML patients with available follow-up. Kaplan-Meier survival analysis was performed to evaluate the association between MRC1 SNPs and OS. The results showed that rs2253120 was associated with OS under the recessive model (*p* = 0.033; **Figure 2B**), and rs691005 was associated with OS under the dominant model (*p* = 0.020; **Figure 2C**). In contrast, rs2253120 did not reach statistical significance under the co-dominant model (*p* = 0.068; **Figure 2A**). Under the recessive model of MRC1 rs2253120, patients with the AA genotype had better OS compared to those with GG+GA genotypes. Under the dominant model of MRC1 rs691005, patients with TT genotype had better OS compared to those with TC+CC genotypes.

Patients aged ≥60 years had notably shorter OS than those aged < 60 years (*p* < 0.001). Patients with a WBC count ≥100×10^9^/L had significantly shorter OS than those with a WBC count <100×10^9^/L (*p* < 0.001). Patients not receiving chemotherapy had significantly shorter OS than those who received treatment (*p* < 0.001). Moreover, OS was significantly shorter in patients with adverse or intermediate risk stratifications compared to those with favorable risk stratifications (*p* < 0.001).

### MRC1 rs2253120 is associated with the prognosis of AML patients

Multivariable Cox proportional hazards regression models were constructed to evaluate the associations of MRC1 rs691005 and rs2253120 genotypes with OS after adjustment for established prognostic factors, including age, risk stratification, WBC count, and chemotherapy status (**Supplementary Table 8**; **Figure 2D**). After adjustment, rs691005 was not independently associated with OS (TC/CC vs TT: HR = 1.262, 95% CI = 0.917-1.736, *p* = 0.153), suggesting that the survival difference observed in Kaplan-Meier analysis may be partly explained by its association with adverse-risk stratification rather than an independent effect on mortality. In contrast, the AA genotype of MRC1 rs2253120 remained associated with improved OS under the recessive model (HR = 0.364, 95% CI = 0.134-0.990, *p* = 0.048).

### MRC1 rs2253120 is associated with MRC1 expression and clinical outcome in AML

**Figure 3:**
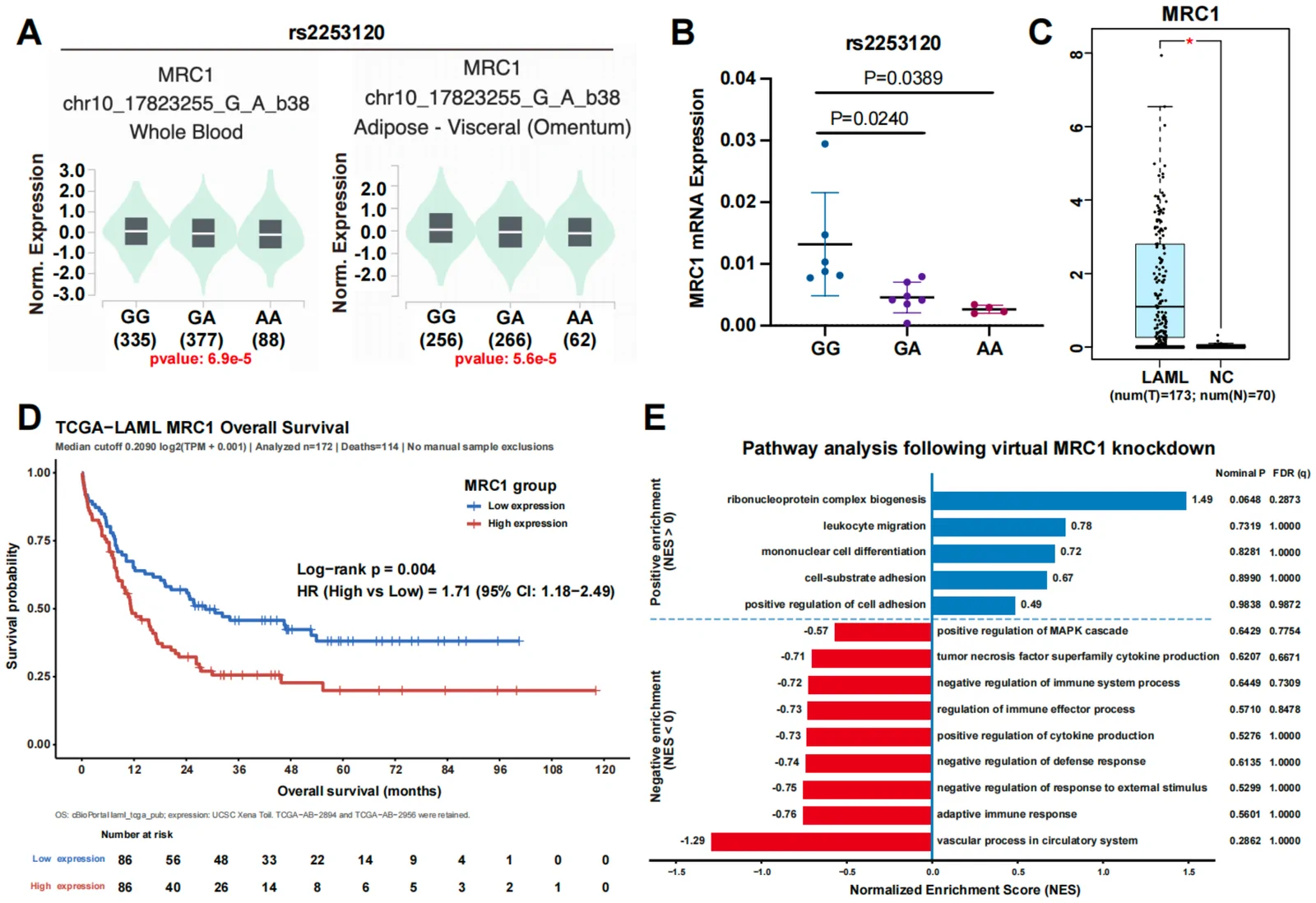
Integrated functional, clinical, and computational characterization of MRC1 in AML. **(A)** Genotype-expression associations for MRC1 rs2253120 in whole blood and visceral adipose tissue from the GTEx database. Violin plots show normalized MRC1 expression across the GG, GA, and AA genotypes. **(B)** Genotype-stratified MRC1 mRNA expression in primary CD34^+^ AML samples. Expression levels were compared among patients carrying the GG (*n* = 6), GA (*n* = 7) and AA (*n* = 4) genotypes. The expression data were normalized to the expression of GAPDH, t-test. Data are presented as mean ± SD. **(C)** Comparison of MRC1 expression between TCGA-LAML samples and normal controls *p<0.05. **(D)** Kaplan-Meier analysis of OS according to MRC1 expression in the TCGA-LAML cohort. Patients were divided into high- and low-expression groups using the median expression value. **(E)** GSEA pathway analysis following virtual MRC1 knockdown in single-cell transcriptomic data.

As rs2253120 showed a consistent association across clinical and survival analyses, subsequent eQTL and genotype-stratified expression analyses focused on rs2253120 to further explore the potential biological basis underlying this association. We first examined whether rs2253120 was associated with MRC1 expression. In GTEx, the rs2253120 A allele was significantly associated with lower MRC1 expression in whole blood, with a concordant association also observed in visceral adipose tissue (whole blood, *p* = 6.9×10^-5^; visceral adipose tissue, *p* = 5.6×10^-5^; **Figure 3A**). These findings suggest that rs2253120, or a functional variant in linkage disequilibrium with it, may be involved in the regulation of MRC1 expression. A similar genotype-dependent pattern was observed in primary AML bone marrow CD34^+^ cells. Compared with the GG group, MRC1 expression was lower in patients with the GA genotype (*p* = 0.0240) and in those with the AA genotype (*p* = 0.0389), with the lowest expression observed among AA carriers (**Figure 3B**).

We next used publicly available transcriptomic datasets to assess MRC1 expression and its prognostic relevance in AML. MRC1 expression was higher in AML samples than in normal controls (173 AML samples and 70 normal controls; **Figure 3C**). In the TCGA-LAML cohort, patients with high MRC1 expression had significantly shorter OS than those with low expression (log-rank *p* = 0.004; HR = 1.71, 95% CI 1.18-2.49; **Figure 3D**).

Finally, we used a single-cell RNA-sequencing dataset generated by our group (16) to explore the cellular programs associated with MRC1. Virtual MRC1 knockdown followed by gene set enrichment analysis revealed positive enrichment trends in pathways related to ribonucleoprotein complex biogenesis, leukocyte migration, mononuclear-cell differentiation, and cell-substrate adhesion. By contrast, pathways involving MAPK signaling, cytokine production, immune-effector regulation, adaptive immune responses, and vascular processes showed negative enrichment trends (**Figure 3E**). However, none of these pathway-level changes remained statistically significant after FDR correction. Therefore, these findings should be interpreted as exploratory and hypothesis-generating, providing potential insights into cellular programs associated with MRC1 expression that require further experimental validation.

Taken together, these analyses showed a consistent directional relationship among rs2253120 genotype, MRC1 expression, and clinical outcome. Carriage of the A allele was associated with lower MRC1 expression, whereas high MRC1 expression was associated with poorer OS. The single-cell virtual knockdown analysis further identified several myeloid and immune-related programs that may be associated with MRC1, although experimental validation will be required.

## Discussion

In this study, either rs691005 nor rs2253120 was associated with AML susceptibility or complete remission after two treatment cycles. The two variants were, however, related to different aspects of the subsequent clinical course. rs691005 was associated with less favorable risk stratification, and patients carrying TC or CC had shorter OS than those with the TT genotype in Kaplan-Meier analysis. rs2253120 showed a different pattern that it was not associated with baseline risk stratification or early remission, but patients with the AA genotype had better OS under the recessive model. The association between rs2253120 genotype and MRC1 expression in primary AML samples provided additional support for this survival finding. Notably, a previous study systematically investigating immunosuppression-related gene polymorphisms in AML reported that MRC1 variants were not significantly associated with AML susceptibility or baseline clinical characteristics (17). While these findings suggested a limited role of MRC1 variants in AML susceptibility and disease presentation, their potential prognostic significance and underlying biological relevance remained largely unexplored. In the present study, we integrated clinical genetic analyses, eQTL data, MRC1 expression validation in primary AML samples, public transcriptomic datasets, and single-cell analysis to extend the clinical implications of MRC1 genetic variation from disease susceptibility and baseline characteristics to patient prognosis and potential biological mechanisms. Collectively, these findings suggest that MRC1 genetic variants may be more relevant to AML progression and clinical outcomes than to disease initiation, potentially by modulating MRC1 expression and immune-related processes within the leukemic microenvironment.

The rs691005 survival result should be interpreted in the context of its association with risk stratification. The shorter survival observed among C-allele carriers may reflect, at least in part, a higher proportion of adverse-risk disease rather than an independent effect of the variant on mortality. Consistent with this interpretation, multivariable Cox regression analysis adjusting for age, risk stratification, white blood cell count, and chemotherapy status showed that rs691005 was not independently associated with overall survival (TC/CC vs TT: HR = 1.262, 95% CI = 0.917-1.736, *p* = 0.153). The lack of an association with CR after two treatment cycles also argues against a simple relationship with resistance to induction therapy. Recent single-cell and spatial studies have shown that durable antileukemia responses depend on coordinated interactions between cytotoxic T cells and other immune populations in the bone marrow, rather than on leukemia-intrinsic characteristics alone (18). An inherited variant could therefore be related to later disease behavior without having a measurable effect on early morphologic remission. Based on the current data, rs691005 is better considered a potential marker associated with unfavorable AML biology rather than an independent prognostic factor, unless its prognostic value is confirmed in larger cohorts with comprehensive adjustment.

The evidence for rs2253120 was broadly consistent across the clinical and expression analyses. Although the variant was not associated with cytogenetic characteristics, risk stratification, or complete remission after two treatment cycles, AA homozygotes had better overall survival than GG/GA carriers. This association remained significant after adjustment for age, risk stratification, chemotherapy, and white blood cell count, with an adjusted HR of 0.364 (95% CI, 0.134-0.990; *p* = 0.048). Sensitivity analyses using Firth penalized Cox regression yielded a similar association under the recessive model (HR = 0.409, 95% CI, 0.135-0.936; *p* = 0.032), supporting the robustness of the observed effect despite the relatively small number of AA homozygotes. However, the additive genetic model did not reach statistical significance (HR = 0.801, 95% CI, 0.613-1.047; *p* = 0.104), suggesting that the potential survival association may be primarily driven by the AA homozygous subgroup rather than a simple allele-dose effect. Thus, rs2253120 AA homozygosity was associated with improved OS. However, given the limited number of AA homozygotes and the borderline confidence interval, this finding requires validation in larger independent cohorts. The expression analyses provided biological context for this survival association. In GTEx, the rs2253120 A allele was associated with lower MRC1 expression. Because eQTL associations detected in bulk tissues can be influenced by cellular composition (19), we also examined MRC1 expression in genotype-stratified primary AML bone marrow CD34^+^ cells and observed the same directional pattern. Meanwhile, due to the limited availability of healthy samples with matched genotype and MRC1 expression data, we were unable to perform a well-powered analysis of genotype-dependent MRC1 expression in healthy individuals. Therefore, it remains unclear whether the regulatory effect of rs2253120 on MRC1 expression represents a general physiological regulatory mechanism or an AML-specific association. In a separate TCGA-LAML cohort, high MRC1 expression was associated with poorer overall survival. Across these analyses, the AA genotype was associated with lower MRC1 expression and favorable survival, whereas high MRC1 expression was associated with an adverse outcome. Although this directional consistency supports a possible biological link, it is not yet fully certain that the reduction in MRC1 expression mediates a survival advantage related to the AA genotype.

The association of rs2253120 with overall survival, but not with remission after two treatment cycles, suggests that its clinical relevance may be related more closely to long-term disease control than to the initial treatment response. One possible explanation involves the bone marrow microenvironment, which can support the survival of AML cells and alter drug sensitivity. Macrophage reprogramming has been proven to reduce this protective effect and restore sensitivity to venetoclax and midostoin (20, 21). These findings illustrate how microenvironmental factors may affect survival without significantly influencing early remission. In this context, high MRC1 expression may reflect the monocyte or macrophage-related state associated with immunosuppression and therapeutic resistance. Monocyte-like AML cells can express MRC1-containing immunomodulatory programs that suppress T-cell activity, and increased numbers of CD206-positive myeloid cells and higher MRC1 expression have been associated with adverse outcomes (2, 21–23). However, these observations do not indicate that MRC1 directly drives this state or that rs2253120 directly regulates MRC1 expression.

Recent advances in virtual-cell modeling and AI-assisted single-cell analysis have shown that computational approaches can be used to simulate cellular responses to genetic or transcriptional perturbations and to generate testable biological hypotheses from large-scale datasets (24–26). However, these approaches remain exploratory and require experimental validation before causal relationships can be established. In our study, virtual MRC1 knockdown was used as an exploratory analysis to identify potential MRC1-associated cellular processes rather than to demonstrate a direct mechanistic effect. Using a single-cell RNA-sequencing dataset generated by our group, virtual reduction of MRC1 expression suggested changes in pathways related to myeloid differentiation, migration, adhesion, MAPK signaling, cytokine production, and immune-effector regulation. These findings are consistent with the known involvement of myeloid cells in immune regulation and interactions within the bone marrow microenvironment. Previous studies showing that cytokine-mediated feedback can enhance AML-directed T-cell activity provide additional context for these observations (27). Nevertheless, none of the identified pathways remained significant after FDR correction, and these results should therefore be interpreted cautiously. One possible explanation is that the analysis was based on a limited number of MRC1-expressing bone marrow cells, and the sparsity of single-cell transcriptomic data may have reduced the ability to detect robust pathway-level changes. Larger single-cell datasets with more MRC1-positive cells, together with experimental perturbation studies, will be needed to further define the cellular processes associated with MRC1.

Several study-wide limitations should also be considered. Because complete clinical and molecular data were not available for all participants, the analyses were based on partially overlapping patient subsets. In addition, the number of variants, multiple genetic models, and clinical outcomes examined increased the possibility of chance-positive findings. Moreover, the single-center retrospective design and limited clinical sample size may have introduced potential selection bias and restricted the statistical power and comprehensiveness of the multivariate analyses. These results therefore require confirmation in larger independent cohorts. Despite the limitations, the study extends beyond a conventional candidate-SNP analysis. It combines case and control data, detailed clinical phenotypes, survival analysis, public eQTL annotation, genotype-stratified expression measurements in primary AML samples, independent transcriptomic survival analysis, and single-cell computational perturbation. Similar integrative approaches have used single-cell data to define cellular states and then linked those states to treatment response and outcome in larger transcriptomic cohorts (28). Such cross-platform analyses can place single-cell observations in a clinically relevant setting.

Overall, the two MRC1 variants were associated with different features of the AML clinical course rather than with disease susceptibility or early treatment response. rs691005 was associated with less favorable risk stratification and shorter OS in Kaplan-Meier analysis. By contrast, rs2253120 AA homozygosity was associated with lower MRC1 expression and was independently associated with improved overall survival after multivariable adjustment. The consistency of the genotype, expression, and survival findings supports a meaningful association between rs2253120 and AML outcome, but neither mediation nor causality has been established. Because SNPs are inherited genetic variants that can generally be assessed using genomic DNA from various biological samples, including blood, buccal swabs, or skin samples, genotyping approaches are technically accessible and may have potential for future clinical application following further validation. The prospective evaluation of this germline polymorphism in clinical cohorts may facilitate the development of genomics-based prognostic models for patients with AML. In future studies, functional approaches, including CRISPR-based genome editing and other experimental technologies, will be employed to validate key SNPs and elucidate the molecular and cellular mechanisms by which these variants may influence AML pathogenesis, disease progression, and clinical outcomes.

## Conclusions

In this study, MRC1 rs691005 was associated with less favorable AML risk stratification and shorter overall survival, whereas rs2253120 AA homozygosity was independently associated with improved overall survival. We also observed a consistent directional relationship among rs2253120 genotype, MRC1 expression, and clinical outcome: carriage of the A allele was associated with lower MRC1 expression, while high MRC1 expression was associated with poorer survival. These findings suggest that inherited variation at the MRC1 locus may contribute to differences in AML clinical behavior, although validation in larger independent cohorts and direct functional studies is still required.

## Data Availability

The datasets presented in this study can be found in online repositories. The names of the repository/repositories and accession number(s) can be found in the article/**Supplementary Material**.
