# Supplementary material for: Associations of polymorphisms in the myeloid immunoregulatory receptor gene MRC1 with expression and prognosis in acute myeloid leukemia
Source: Front Immunol. 2026 Sep 7;17:1949691. doi: 10.3389/fimmu.2026.1949691 (PMC13590749; doi:10.3389/fimmu.2026.1949691)
Supplement: Supplementary file 1 [file Table1.docx]

**Supplementary Table 1.** Association between MRC1 gene polymorphism and WBC in AML

| Gene | SNP | Model | Genotype | WBC<  100×10^9^ /L | WBC≥  100×10^9^/L | 𝜒2 test  *p* value | OR (95% CI) | Adjusted *p* value |  |
| --- | --- | --- | --- | --- | --- | --- | --- | --- | --- |
| MRC1 | **rs691005** | Co-dominant | TT | 98 | 24 | 0.932 |  |  |  |
|  |  |  | CT | 93 | 21 |  | 1.015  (0.523-1.970) | 0.965 |  |
|  |  |  | CC | 34 | 9 |  | 1.019  (0.427-2.432) | 0.967 |  |
|  |  | Dominant | TT | 98 | 24 | 0.906 |  |  |  |
|  |  |  | CT+CC | 127 | 30 |  | 1.016  (0.554-1.863) | 0.959 |  |
|  |  | Recessive | TT+CT | 191 | 45 | 0.776 |  |  |  |
|  |  |  | CC | 34 | 9 |  | 1.012  (0.447-2.289) | 0.977 |  |
|  | **rs2253120** | Co-dominant | GG | 129 | 36 | 0.335 |  |  |  |
|  |  |  | GA | 84 | 17 |  | 0.766  (0.401-1.464) | 0.420 |  |
|  |  |  | AA | 12 | 1 |  | 0.235  (0.029-1.906) | 0.175 |  |
|  |  | Dominant | GG | 129 | 36 | 0.210 |  |  |  |
|  |  |  | GA+AA | 96 | 18 |  | 0.684  (0.364-1.285) | 0.237 |  |
|  |  | Recessive | GG+GA | 213 | 53 | 0.474 |  |  |  |
|  |  |  | AA | 12 | 1 |  | 0.256  (0.032-2.061) | 0.201 |  |
| SNP, single nucleotide polymorphisms; OR, odds ratio; CI, confidence interval. | | | | | | | | | |

**Supplementary Table 2.** Association between MRC1 gene polymorphism and PLT in AML

| Gene | SNP | Model | Genotype | PLT>  50×10^9^/L | PLT≤  50×10^9^/L | 𝜒2 test  *p* value | OR (95% CI) | Adjusted *p* value |
| --- | --- | --- | --- | --- | --- | --- | --- | --- |
| MRC1 | **rs691005** | Co-dominant | TT | 53 | 69 | 0.523 |  |  |
|  |  |  | CT | 42 | 72 |  | 1.252  (0.737-2.128) | 0.406 |
|  |  |  | CC | 19 | 24 |  | 0.997  (0.492-2.022) | 0.993 |
|  |  | Dominant | TT | 53 | 69 | 0.439 |  |  |
|  |  |  | CT+CC | 61 | 96 |  | 1.172  (0.721-1.907) | 0.521 |
|  |  | Recessive | TT+CT | 95 | 141 | 0.630 |  |  |
|  |  |  | CC | 19 | 24 |  | 0.899  (0.462-1.748) | 0.754 |
|  | **rs2253120** | Co-dominant | GG | 69 | 96 | 0.211 |  |  |
|  |  |  | GA | 37 | 64 |  | 1.226  (0.732-2.052) | 0.438 |
|  |  |  | AA | 8 | 5 |  | 0.479  (0.148-1.554) | 0.220 |
|  |  | Dominant | GG | 69 | 96 | 0.695 |  |  |
|  |  |  | GA+AA | 45 | 69 |  | 1.094  (0.669-1.788) | 0.720 |
|  |  | Recessive | GG+GA | 106 | 160 | 0.120 |  |  |
|  |  |  | AA | 8 | 5 |  | 0.446  (0.139-1.427) | 0.174 |
| SNP, single nucleotide polymorphisms; OR, odds ratio; CI, confidence interval. | | | | | | | | |

**Supplementary Table 3.** Association between MRC1 gene polymorphism and HGB in AML

| Gene | SNP | Model | Genotype | HGB ≥  60 g/L | HGB <  60 g/L | 𝜒2 test  *p* value | OR (95% CI) | Adjusted *p* value |
| --- | --- | --- | --- | --- | --- | --- | --- | --- |
| MRC1 | **rs691005** | Co-dominant | TT | 108 | 14 | 0.136 |  |  |
|  |  |  | CT | 92 | 22 |  | 1.885  (0.906-3.922) | 0.090 |
|  |  |  | CC | 39 | 4 |  | 0.784  (0.243-2.532) | 0.685 |
|  |  | Dominant | TT | 108 | 14 | 0.229 |  |  |
|  |  |  | CT+CC | 131 | 26 |  | 1.541  (0.765-3.105) | 0.226 |
|  |  | Recessive | TT+CT | 200 | 36 | 0.306 |  |  |
|  |  |  | CC | 39 | 4 |  | 0.565  (0.189-1.688) | 0.307 |
|  | **rs2253120** | Co-dominant | GG | 145 | 20 | 0.434 |  |  |
|  |  |  | GA | 83 | 18 |  | 1.571  (0.785-3.146) | 0.202 |
|  |  |  | AA | 11 | 2 |  | 1.319  (0.269-6.480) | 0.733 |
|  |  | Dominant | GG | 145 | 20 | 0.204 |  |  |
|  |  |  | GA+AA | 94 | 20 |  | 1.541  (0.786-3.020) | 0.208 |
|  |  | Recessive | GG+GA | 228 | 38 | 1.000 |  |  |
|  |  |  | AA | 11 | 2 |  | 1.104  (0.231-5.265) | 0.902 |
| SNP, single nucleotide polymorphisms; OR, odds ratio; CI, confidence interval. | | | | | | | | |

**Supplementary Table 4.** FDR correction for AML susceptibility

| SNP | Model | Nominal *p* | FDR-adjusted *p* |
| --- | --- | --- | --- |
| rs691005 | Co-dominant | 0.788 | 0.943 |
|  | Dominant | 0.943 | 0.943 |
|  | Recessive | 0.503 | 0.943 |
| rs2253120 | Co-dominant | 0.411 | 0.943 |
|  | Dominant | 0.191 | 0.943 |
|  | Recessive | 0.915 | 0.943 |

**Supplementary Table 5.** FDR correction for AML risk stratification

| SNP | Model | Nominal *p* | FDR-adjusted *p* |
| --- | --- | --- | --- |
| rs691005 | Co-dominant | 0.002 | **0.006** |
|  | Dominant | <0.001* | **0.006** |
|  | Recessive | 0.083 | 0.166 |
| rs2253120 | Co-dominant | 0.543 | 0.543 |
|  | Dominant | 0.299 | 0.449 |
|  | Recessive | 0.493 | 0.543 |

*P<0.001 was replaced by 0.001 for FDR calculation.

**Supplementary Table 6.** FDR correction for induction chemotherapy response

| SNP | Model | Nominal *p* | FDR-adjusted *p* |
| --- | --- | --- | --- |
| rs691005 | Co-dominant | 0.394 | 0.944 |
|  | Dominant | 0.553 | 0.944 |
|  | Recessive | 0.355 | 0.944 |
| rs2253120 | Co-dominant | 0.944 | 0.944 |
|  | Dominant | 0.914 | 0.944 |
|  | Recessive | 0.665 | 0.944 |

**Supplementary Table 7.** Sensitivity analyses of the association between rs2253120 and overall survival in non-M3 AML

| Analysis | Genetic model | Genotype | Patients (n) | Events (n) | HR | 95% CI | *p* value |
| --- | --- | --- | --- | --- | --- | --- | --- |
| Conventional Cox regression | Recessive Model | GG/GA | 231 | 167 | Reference | — | — |
|  |  | AA | 11 | 4 | 0.364 | 0.134–0.990 | **0.048** |
| Firth penalized  Cox regression | Recessive Model | GG/GA | 231 | 167 | Reference | — | — |
|  |  | AA | 11 | 4 | 0.409 | 0.135–0.936 | **0.032** |
| Conventional Cox regression | Additive Model | GG | 140 | 105 | 0.801 (per A allele) | 0.613–1.047 | 0.104 |
|  |  | GA | 91 | 62 |  |  |  |
|  |  | AA | 11 | 4 |  |  |  |
| Firth penalized  Cox regression | Additive Model | GG | 140 | 105 | 0.806 (per A allele) | 0.613–1.045 | 0.105 |
|  |  | GA | 91 | 62 |  |  |  |
|  |  | AA | 11 | 4 |  |  |  |

**Notes:** All models were adjusted for age, risk stratification, white blood cell count, and chemotherapy status.

The recessive model compared AA with GG/GA carriers. The additive model coded GG = 0, GA = 1, and AA = 2; the reported HR represents the effect per additional A allele.

Firth-model confidence intervals and P values were obtained from the profile penalized likelihood. The conventional Cox model used the Breslow approximation for tied event times.

The survival cohort included 242 patients and 171 deaths. Eleven patients had the AA genotype, of whom four died during follow-up.

**Supplementary Table 8.** Multivariable Cox regression analysis of overall survival according to rs691005 dominant model

| Variable | Category | Number (n) | HR (95% CI) | *p* value |
| --- | --- | --- | --- | --- |
| rs691005 | TT | 107 | Reference | — |
|  | TC/CC | 135 | 1.262 (0.917–1.736) | 0.153 |
| Age | <60 years | 178 | Reference | — |
|  | ≥60 years | 64 | 1.461 (1.014–2.104) | **0.042** |
| Risk stratification | Favorable | 47 | Reference | — |
|  | Intermediate | 110 | 2.184 (1.316–3.627) | **0.003** |
|  | Adverse | 85 | 2.891 (1.707–4.897) | **<0.001** |
| Chemotherapy | No | 42 | Reference | — |
|  | Yes | 200 | 0.414 (0.279–0.615) | **<0.001** |
| WBC count | <100 × 10⁹/L | 191 | Reference | — |
|  | ≥100 × 10⁹/L | 51 | 1.750 (1.222–2.506) | **0.002** |

**Note:** Multivariable Cox proportional hazards regression model was adjusted for age, risk stratification, white blood cell count, and chemotherapy status
